# Gaining Perspective on What We've Lost: The Reliability of Encoded Anecdotes in Historical Ecology

**DOI:** 10.1371/journal.pone.0043386

**Published:** 2012-08-24

**Authors:** Dalal Al-Abdulrazzak, Robin Naidoo, Maria Lourdes D. Palomares, Daniel Pauly

**Affiliations:** 1 Fisheries Centre, University of British Columbia, Vancouver, British Columbia, Canada; 2 Conservation Science Program, WWF-US, Washington, D.C., United States of America; Phillip Island Nature Parks, Australia

## Abstract

Historical data are essential in fisheries management and conservation, especially for species that suffered significant population declines prior to ecological data collection. Within the field of historical marine ecology, studies have relied on anecdotal evidence, such as written accounts by explorers and interviews of different generations of resource users, to demonstrate the former abundance of certain species and the extent of their ranges. Yet, do we all agree on how these anecdotes are interpreted? This study examines the way that different people interpret anecdotes extracted from historical narratives. We outsource a survey to 50 randomly selected people using Amazon Mechanical Turk (www.mturk.com) and ask them to ‘code’ historical anecdotes based on their perceived abundance of species. We perform intercoder reliability tests to show that people's perceptions of historical anecdotes are generally consistent. The results speak to the reliability of using people's perceptions to acquire quantitative data, and provide novel insights into the use of anecdotal evidence to inform historical ecology.

## Introduction

Marine ecology is a relatively young science, with few descriptive studies extending back for more than a century. Thus until recently, marine ecologists have tried to explain patterns of distribution and abundance based on short-term experiments and ‘real time’ observations [1]. This shortsightedness has resulted in studying ecological states that were already degraded, yet believing they were ‘pristine’. This is manifest in many examples throughout the world, but most notably the collapse of Jamaica's coral reefs, which were thought to be amongst the healthiest and most well-studied reefs at the time [2].

The situation is even worse for fisheries science, a discipline that has long suffered from a lack of historical reflection. In 1995 Pauly coined the term “shifting baseline syndrome” to describe the incremental lowering of standards, with respect to fisheries, so that each new generation redefines what is ‘natural’ according to personal experience and looses sight of how the environment used to be [3]. These shifting ecological baselines have resulted in lowered expectations for the natural abundances of marine animals and the ecosystem services they provide [4], [5], [6], [7]. Populations of fishes, large vertebrates, marine mammals, and certain invertebrates thought to persist in “healthy” numbers today may, in fact, be small fractions of their historical abundance. Historical accounts from the 1700s and early 1800s mention seas teeming with large fish, yet accounts like these are virtually unheard of today.

Pauly's call for the incorporation of earlier anecdotal knowledge into traditional ecological studies prompted a body of literature based on the premise that historical anecdotes, rooted in human experience, can provide powerful insights into long-term changes in marine ecosystems [3]. These studies in historical marine ecology have uncovered surprising findings about the structure and function of past ecosystems, and have affected our understanding of species declines, trends in global fisheries, and overall ecological integrity [4], [7], [8], [9], [10]. Results of these analyses have shown that human impacts in coastal ecosystems have been far more substantial than previously thought, and have deepened our understanding of the connection between social history and marine ecosystems.

While historical perspectives are increasingly necessary to understand marine ecosystem structure and function, the majority of species-associated historical data prior to the second half of the 20^th^ century remains anecdotal [11], [12], [13], raising questions about the validity of findings. Deriving quantitative insights from qualitative historical narratives often requires a form of content analysis. One such method is coding, or the categorization of large amounts of narratives to identify common patterns or themes. This method is most often used in social sciences, where qualitative surveys or interviews are coded to draw patterns in subjective experiences [14]. Although the majority of coding studies have relied on *ad hoc* categories based on the judgment and objectives of the researcher, studies with meaningful categories from which to code accounts can assimilate seemingly disparate events or objects to identify new patterns [15].

Within historical ecology the majority of coding studies have reconstructed ecological trajectories of species over time by applying consistent criteria to code anecdotes [10], [11], [12], [16], [17]. Yet, because these reconstructions were based on a single person's perception of a set of historical anecdotes or many people coding different anecdotes, the external validity of these results cannot be evaluated.

To overcome criticism that the interpretation of qualitative anecdotal data in historical ecology is overly subjective, we test whether people perceive similar species' abundances from historical anecdotes. We use intercoder reliability testing, a standard measure of consistency, to determine the degree to which independent coders agree on the ranking of historical anecdotes using the same coding scheme. Similar to the subjectivity encountered in fish age interpretation by otolith readers [18], [19], repeated readings of historical anecdotes by different people can verify that the original vision of authors remains implicit, and therefore whether the conclusions drawn are valid.

## Methods

### Ethics statement

This study was approval by the Behavioral Research ethics Board of the University of British Columbia. Written consent was obtained by completion of the questionnaire. The University of British Columbia Behavioural Research Ethics Board (BREB) procedures and Guidance Notes comply with the second edition of the Tri-Council Policy Statement (TCPS) on ‘Ethical Conduct for Research Involving Humans’ (TCPS2). The UBC BREB operates under the authority of UBC Policy 89 on Research and Other Studies Involving Human Subjects.

### Survey

We extracted 50 anecdotal accounts (defined here as informal—often brief—earlier accounts of species' abundances) of marine organisms from historical texts on the Persian Gulf, the Falkland Islands, and Raja Ampat (Papua, Indonesia), ranging in date from 1330 to 1940 (Table 1). Because an anecdote's date may be inferred from certain features, such as the dates and names of people and places, we remove any identifying information. We also excluded passages where the style of language was immediately indicative of the era, as to ensure that coders were not positively biased towards passages that were perceived as older (i.e., interpreting greater abundance from older anecdotes, and less from newer anecdotes).

**Table 1:** Examples of historical anecdotes used in the coding survey. Identifying features are replaced with “-------”.

Reference	Passage
Ibn Battutah [31]	“Most of the fish in it are the species called sardin, which are extremely fat there. It is a strange fact that their beasts have their sole fodder these sardines, and likewise their flocks, and I have never seen this any other place.”
Pernety [32]	“We did not catch any beautiful shell-fish here; the only one deserving notice was a helmet shell, which was at least eight inches in diameter.”
Streeter [33]	“Among the dangers of the pearler in the ------- the dreaded saw fish may be mentioned as the chief enemy. This shark like creature is furnished with a formidable weapon in the shape of a flat projecting snout reaching a length of perhaps six feet and armed along its edges with strong toothlike spines. In the presence of such a terrific weapon the diver is almost powerless and instances are recorded in which the poor fellows have been completely cut in two.”
Villiers [34]	“Fine edible fish seemed extraordinarily numerous off that coast, and all day long the fishermen were landing their heavy catches through the surf”

We created a multi-level species abundance classification scheme (Table 2) based on systems used in Palomares *et al*. and Pandolfi *et al.* [10], [12], [16]. For each of the 50 anecdotal accounts, participants were asked to select one of five ‘species abundance descriptors,’ based on their perceived abundance of the species described in the passage. Although species' abundances are typically relative to their trophic level (i.e., predators are often less abundant than prey), the criteria describe relative depletion of species, rather than absolute values in species abundance. We make this distinction because it is possible to have a small population of highly productive small prey animals supporting a relatively high biomass of larger predators [20].

**Table 2:** Coding criteria of perceived species' abundances following the ranking system applied in Palomares *et al*. (2007, 2006) and Pandolfi *et al.* (2003).

Species Abundance Descriptor	Criteria for Classification
Abundant	Account lacks any evidence of human use or reduced species abundance
Common	Account describes some human use, but no evidence of reduced species abundance
Present	Account describes some human use and evidence of reduced species abundance
Rare	Account describes extreme human use and severely reduced species abundance
Absent	Species no longer in existence

We outsourced our survey to 50 people using Amazon Mechanical Turk (www.mturk.com), a crowd-sourcing Internet marketplace that coordinates the supply and demand of tasks requiring human intelligence. Studies have shown that micro-task markets are useful for studies that require access to a large user pool for subjective information gathering [21]. Since the so-called ‘Turks’ are drawn from a wide range of users (virtually anyone connected to the internet), they represent a diverse range of perspectives and therefore complimented the goals of our study.

Since there is no incorrect way to answer our survey, we attempted to reduce the likelihood of Turks ‘gaming’ the system (i.e., providing nonsense answers in order to decrease their time spent on the task and thus increase their rate of pay) by planting a ‘trick’ question within the survey to determine the authenticity of responses. We removed those surveys where Turks did not answer the trick question correctly. We also reviewed the time taken to complete each survey and removed surveys that were submitted in 10 minutes or less, as we considered it unlikely that respondents could reliably answer in this time.

Furthermore, because there are 39 agreement indices and no consensus on the best index to determine intercoder reliability, we performed three common reliability tests for categorical rankings to determine the proportion of variance in rankings due to between-subject variability in the true scores: 1) Intraclass Correlation Coefficient (ICC), describes how strongly units in the same group resemble each other, while 2) Fleiss Kappa and 3) Finn-Coefficient which describe the reliability of agreement between a fixed number of coders assigning categorical rankings [22], [23]. Coefficient values range from 0 to 1, with 1 representing perfect agreement. Although there is no minimum acceptable level of reliability for all indices, coefficients of .80 or higher are acceptable in most cases, and lower levels are acceptable for more conservative indices such as the Fleiss Kappa [22].

## Results

Of the 50 surveys solicited, 4 coders failed to answer the ‘trick’ question correctly, and 6 coders submitted the survey in less than 10 minutes, resulting in a total of 40 surveys that were suitable for analysis.

**Figure 1:**
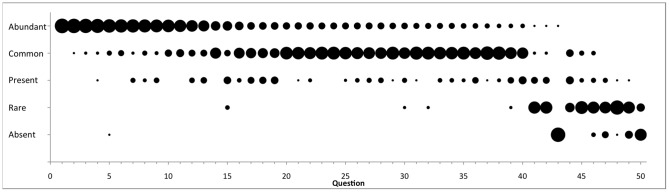
Summary of responses across all 50 questions. Questions are ordered on the x-axis by decreasing frequency of the most abundant descriptor ranking (i.e. “Abundant” to “Absent”). Circle size is proportional to frequency of response.

We graph the results of all responses across questions using a modified dot plot to show the level of agreement among the respondents across all questions (Figure 1). For each question, dot size is proportional to response frequency: the larger the dot, the more frequently a species abundance descriptor was selected by respondents and therefore the greater the level of agreement. Questions with lower levels of agreement are indicated by an even distribution of smaller dots across species abundance indicators. We order questions in decreasing order of response frequency of the “Abundant” descriptor, with ties broken by decreasing order of the “Common” descriptor, and further ties broken by subsequent descriptors. “Common” and “Abundant” were the most commonly selected species descriptors (41% and 32% of total responses, respectively), while the average (weighted by response frequency) number of descriptors selected per question was 1.45 (minimum = 1, maximum = 2.35).

The results of both the ICC test and Finn-Coefficient indicate strong intercoder reliability (ICC = 0.743; Finn-Coefficient = 0.834; Table 3), while the Fleiss Kappa indicates moderate reliability (Kappa = 0.407; Table 3). Although the Fleiss Kappa value is lower than the other two indices, it does not necessarily point to low levels of agreement, because unlike the other two indices the Fleiss Kappa considers the prevalence of rankings, indicating an uneven distribution of categorical rankings [24]. When taken in context of the high levels of agreement by the other two indices, and the fact that 73% of total responses were “Common” and “Abundant,” the low levels of Kappa are most likely an artifact of rarely chosen rankings.

**Table 3:** Statistical summary for three commonly used intercoder reliability tests.

Test	Value	P-Value	Confidence interval
Intraclass correlation Coefficient (ICC)	0.743	p<0.001	0.665–0.819
Fleiss' Kappa	0.407	p<0.001	N/A
Finn Coefficient	0.835	p<0.001	N/A

## Discussion

Intercoder reliability, or the extent to which independent coders evaluate a characteristic of a subject (anecdotes in this case) and reach the same conclusion, is a critical component of content analysis [22], [25]. Reliable coding demonstrates replicability, a fundamental component of scientific research. Here, we show that text coding, a method commonly used in historical marine ecology, can achieve high levels of intercoder reliability, challenging the notion that anecdotal evidence is irrelevant [26], [27]. In this way, intercoder reliability can be used as a proxy for the validity of conclusions drawn from anecdotal data.

Humans, possessing both consciousness and culture, are predisposed to see or miss things, count or ignore them [28]. While the precision and clarity of individual historical accounts may vary, using many anecdotes that exhibit similar ecological trends greatly increases confidence in the results [4], [21]. Anecdotal evidence, taken in quantity, can overcome the particular biases of individual sources, to produce a rough picture of how ecosystems used to look [4].

Despite the importance of historical baselines in setting recovery and conservation goals, historical data in the form of anecdotes or narratives are not commonly incorporated into existing management contexts [29]. Integrating qualitative information into established quantitative frameworks or standardized assessment protocols is challenging at best [29]. In the absence of quantitative data, coding anecdotal accounts can help overcome the psychological barrier that leads one to believe that no data exist. For example, coding historical accounts may be useful in establishing historical baselines for endangered species such as sawfishes (Pristidae) in the Persian Gulf. Eyewitness accounts by pearl divers in the 18^th^ century suggest sawfishes were once abundant, yet accounts of sawfishes today are extremely rare. Despite the apparent decline in sawfish populations, management plans are stalled by the lack of quantitative data. Establishing intercoder reliability can add legitimacy to studies based on historical anecdotes, facilitating their integration into conservation and management frameworks.

We suggest that future coding studies in historical ecology perform intercoder reliability tests to verify if the particular scale chosen is appropriate; low levels of agreement among coders may suggest weaknesses in research methods, including the possibility of poor category definitions and coder training. High intercoder agreement, on the other hand, strengthens conclusions drawn from anecdotal evidence. In this way, the calibration of people's perceptions of qualitative narratives adds value to anecdotal evidence allowing for the integration of varying data types.

The establishment of high levels of reliability among coders also has the practical benefit of allowing researchers to distribute the coding work among many different coders, thus improving efficiency [25]. Here, we also demonstrate the utility of outsourcing coding tasks using Amazon Turk. Despite their lack of training (and perhaps interest) in the subject, Turks were able to achieve acceptable levels of intercoder reliability. We predict that with some preliminary training, outsourced coding studies can achieve even higher reliability values. Furthermore, we predict that historical ecology researchers (i.e., experts) are likely to generate a more cohesive result due to their disciplinary training devoted to the critical examination of historical sources. Since Turks are composed of a wide range of users, they likely use different sets of criteria in subjective decision-making than expert populations [21].

While the calibration of perspectives is useful, it is important to note that coding allows only for broad inferences in past species abundances. Stripping qualitative narratives of their richness and variety, and transforming them into categorical units, sacrifices historical and/or ecological precision, impeding our ability to make prescriptive statements about the state of past or future ecosystems. Only by incorporating a variety of sources and analytical techniques with expert knowledge can we begin to have a more nuanced perspective to make broad estimates on the general pace and direction of changes in species biodiversity and biomass.

A historical perspective is needed to envision what oceans might have looked like in the past and what they can produce in the future. In the face of limited knowledge, anecdotes serve as useful starting points for ecological studies. If limits are placed on the conclusions drawn, anecdotes can provide rich insights into structure and function of past ecosystems [30]. This contribution suggests that people's perceptions of species' abundances from historical narratives are generally consistent and that intercoder reliability can complement future studies in historical ecology by calibrating perceptions of anecdotal accounts
